# Supplementary material for: Feasibility and acceptability of continuous glucose monitoring in pregnancy for the diagnosis of gestational diabetes: A single-centre prospective mixed methods study
Source: PLoS One. 2023 Sep 27;18(9):e0292094. doi: 10.1371/journal.pone.0292094 (PMC10529558; doi:10.1371/journal.pone.0292094)
Supplement: S1 File — (DOCX) [file pone.0292094.s001.docx]

**S1: Qualitative interview questions asked to the participants after they had completed the study.**

- Why did you decide to participate in this study?
- Do you think glucose testing in pregnancy is important?
- The diagnostic process for gestational diabetes has had to change during the covid-19 pandemic. How do you feel about this?
- What were your first impressions of using the CGM?
- Did you experience any problems or difficulties using the CGM?
- What were your experiences of doing the home OGTT?
- Did you encounter any problems logging the time you started the OGTT?
- Would you recommend this method to other women who need glucose testing in pregnancy?
