# Supplementary material for: Feasibility and acceptability of continuous glucose monitoring in pregnancy for the diagnosis of gestational diabetes: A single-centre prospective mixed methods study
Source: PLoS One. 2023 Sep 27;18(9):e0292094. doi: 10.1371/journal.pone.0292094 (PMC10529558; doi:10.1371/journal.pone.0292094)
Supplement: S2 File — (DOCX) [file pone.0292094.s002.docx]

**S2: Instructions given about the OGTT**

**Covid-19 GestTesting Study Brief instructions for participants v1; 20/4/2020.**

**Instructions for the care of your Continuous Glucose Monitor (CGM) & your home-OGTT (oral glucose tolerance test)**

**Thank you very much for taking part in the Covid19 Gest Testing study.**

**Conducting your home glucose tolerance test – you will only have to do this once.**

1. When we sited your continuous glucose monitor (CGM) we also gave you a glucose drink called Rapilose solution.

2. On day 3 please eat your normal evening meal with your normal drinks. After that, do not eat or drink anything else until your test the following morning. You can still have sips of water but all other drinks should be avoided.

3. On day 4, wake up at your normal time but do not have breakfast. Please take the Rapilose drink at 9am exactly.

4. Please write down the exact time of starting to drink the solution and log this in your CGM receiver (we will show you how to do this). The Rapilose solution should be drunk within 5 minutes and you should make sure it has all been consumed. You should avoid exercise and do not eat again for another 3 hours.

5. Please ensure the sensor is in place during this time because it will measure your blood glucose levels at 0, 1and 2 hours.

6. Day 4 is the best time to do this based on peak sensor accuracy, but days 3-5 would also be satisfactory if more convenient for you.

**Care of your continuous glucose monitor**

- Please keep your receiver near to you (looks a bit like a mobile phone). It should be kept within 6 metres of the sensor that is on your arm for most of the time.
- You can shower or take baths with the sensor on your arm…it is shower proof. You can also do exercise with it on.
- Your sensor should last for 7-10 days. If you notice that your sensor is coming off early (i.e. before 7 days) then use the Tegaderm dressing which is in your study pack OR use plasters to keep it stuck in place.
- After the sensor has been on your arm for 7-10 days it will stop working and you will be able to remove it. We need all pieces of the equipment back to access your data.
- Removing your sensor – you will be able to remove the sensor just like you would remove a sticky plaster. When you have removed it please place it back in the box. Place all the study equipment back in your study pack.

Please email the study team on: add-tr.digeststudy@nhs.uk with any questions you have about this and also **to let us know when you are ready for us to collect your study pack from in a safe place outside your home**. One of the study team will come to collect it. They will not knock at your door as the aim is to maintain non-direct contact as much as possible during the Covid-19 pandemic

**Thank you very much for taking part in the Covid19 Gest Testing study.**
