# Supplementary material for: Feasibility and acceptability of continuous glucose monitoring in pregnancy for the diagnosis of gestational diabetes: A single-centre prospective mixed methods study
Source: PLoS One. 2023 Sep 27;18(9):e0292094. doi: 10.1371/journal.pone.0292094 (PMC10529558; doi:10.1371/journal.pone.0292094)
Supplement: Attachment — Submitted filename: Gest Test Qual Response to reviewers 2023_07_19.docx [file pone.0292094.s003.docx]

Reviewer's Responses to Questions

**Comments to the Author**

1. Is the manuscript technically sound, and do the data support the conclusions?

The manuscript must describe a technically sound piece of scientific research with data that supports the conclusions. Experiments must have been conducted rigorously, with appropriate controls, replication, and sample sizes. The conclusions must be drawn appropriately based on the data presented.

Reviewer #1: Partly

Reviewer #2: Partly

Comments: Thank you for your reviews. We have made the changes recommended below and hope that this has addressed any concerns about the robustness of our findings.

2. Has the statistical analysis been performed appropriately and rigorously?

Reviewer #1: Yes

Reviewer #2: I Don't Know

Comments: This is a qualitative paper with little quantitative analysis. We have made the changes recommended below and hope that this has addressed any concerns about the analysis.

3. Have the authors made all data underlying the findings in their manuscript fully available?

The [PLOS Data policy](https://eur03.safelinks.protection.outlook.com/?url=http%3A%2F%2Fwww.plosone.org%2Fstatic%2Fpolicies.action%23sharing&data=05%7C01%7Cclm70%40universityofcambridgecloud.onmicrosoft.com%7C7cae9e06d43b496d86cd08db66e9845c%7C49a50445bdfa4b79ade3547b4f3986e9%7C1%7C0%7C638216926388362857%7CUnknown%7CTWFpbGZsb3d8eyJWIjoiMC4wLjAwMDAiLCJQIjoiV2luMzIiLCJBTiI6Ik1haWwiLCJXVCI6Mn0%3D%7C3000%7C%7C%7C&sdata=uZn4fZG3wbM2we1HSXqPgxNF1pqv3gA%2Buyyit3uji7o%3D&reserved=0) requires authors to make all data underlying the findings described in their manuscript fully available without restriction, with rare exception (please refer to the Data Availability Statement in the manuscript PDF file). The data should be provided as part of the manuscript or its supporting information, or deposited to a public repository. For example, in addition to summary statistics, the data points behind means, medians and variance measures should be available. If there are restrictions on publicly sharing data—e.g. participant privacy or use of data from a third party—those must be specified.

Reviewer #1: No

Reviewer #2: No

Comments: This is a qualitative paper and excerpts from all interview transcripts are included within the manuscript. There is no additional data file to make available. We would consider making transcripts of interviews available to readers for reasonable requests if participant confidentiality was not compromised (see line 162). We hope that this has addressed any concerns about data availability.

4. Is the manuscript presented in an intelligible fashion and written in standard English?

PLOS ONE does not copyedit accepted manuscripts, so the language in submitted articles must be clear, correct, and unambiguous. Any typographical or grammatical errors should be corrected at revision, so please note any specific errors here.

Reviewer #1: Yes

Reviewer #2: Yes

Comments: Thank you.

5. Review Comments to the Author

Please use the space provided to explain your answers to the questions above. You may also include additional comments for the author, including concerns about dual publication, research ethics, or publication ethics. (Please upload your review as an attachment if it exceeds 20,000 characters)

Reviewer #1: Thank you for doing this research on a significant issue. The use of CGM seems to have a powerful future role in both the diagnosis and the management of GDM.

Comments:

1. There are very few studies on the use of CGM in the diagnosis, which also report their acceptability among women. Please, cite them in the introduction and also make a comparison with your study in the discussion.

Comments: We have added more text to the introduction highlighting these studies and providing more contextual background from the literature (lines 71-86, 376-383).

2. Also, Can you please describe the clinical/ scientific reasoning for masking the CGM data from the clinical decision team?

Comments: CGM is not a validated tool for use for diagnosis or monitoring of gestational diabetes. Although CGM is occasionally used in GDM, at the time of data collection, ~2021, this was uncommon. Masking the data therefore avoids patient anxiety, especially in a situation where clinical targets or diagnostic thresholds have not been determined.

3. There have been separate questions specifically asked to assess whether the process of wearing or removing the device was found to be painful or not. I understand that one participant mentioned it, but that is very important when we assess acceptability. Also, documentation on any side effects.

Comments: We have added more text to the manuscript about acceptability and pain (lines 280-282 and in table 3). We have also added an additional section on side effects which were mentioned by some of the participants (lines 291-297).

Reviewer #2: General comment: This manuscript makes an important contribution to the field of gestational diabetes; suggesting that CGM (potentially combined with OGTT) done at home, is more acceptable than standard of care. However, I have a few concerns about the qualitative aspect of the research. I make a few suggestions and raise a few minor questions below.

Title:
Suggest including setting or location in which study was conducted in the title.

Comments: We have changed the title to clarify this.

Abstract:
Line 33: 'pre-determined interview schedule' is later referred to as 'semi-structured interview questions'. Suggest using consistent terminology throughout. The former seems more in line with a 'structured interview schedule' than a semi-structured interview schedule. This has methodological implications.

Comments: We have now changed this to improve consistency.

Introduction:
Line 53: Suggest including a brief description of what OGTT entails i.e., in standard of care, to better orient the reader to the following two paragraphs.
Line 60: remove 'high risk' from 'high risk ethnicity'. Ethnicity is a risk factor.
Line 75: remove 'the' before 'CGM'.

Comments: We have now addressed these issues. Please see lines 55-57 for additional information.

Participants and methods:
Since these data were collected during the peak of the COVID-19 pandemic, it might be helpful to include a brief description of the setting in which the data were collected. E.g., what, if any, restrictions were in place regarding healthcare access?

Comments: We have now added further information about the setting (lines 100-107).

Study design and participants:
Line 84: The current description of the study design, only describes the 'clinical' side of the study. It does not describe the design of the qualitative study.

Comments: We have now added further information to address your comments (Lines 158-185).

Lines 84-86: Suggest this be moved to a separate section entitled, 'ethics' or 'ethical considerations'. In this section, also include whether participants were reimbursed.

Comments: We have now changed the structure to address your comments (Lines 96-98) and added a sentence about reimbursement (line 116).

Recruitment process:
There is no description of how the 20 participants recruited for the qualitative part of the study were selected. What sampling approach was followed?

Comments: We used voluntary response sampling and have added this to the manuscript (line 160).

Line 92: 'pregnancy with one of more risk factors', do you mean 'pregnancy with one or more risk factors'?

Comments: We have now changed this to address your comments (Line 104).

GDM diagnosis:
Lines 126-127: How does this differ from the diagnostic and processual criteria followed outside of COVID-19 conditions?

Comments: We have now added further information to address your comments (lines 100-107).

Qualitative analysis:

There are two sections in the manuscript entitled 'qualitative analysis'. (See Line 139 and Line 166).

Comments: We have now removed one of these headings and changed it to Qualitative themes (Line 204).

Why were only 20 participants recruited to the qualitative study? Was saturation reached?

Comments: Yes, data saturation was reached. We have now added further information to clarify this (Line 206-207).

Lines 147-149: What kind of thematic analysis was used? Provide references for the approach used. E.g., What framework of acceptability was used to guide analysis?

Comments: We have now added further information to address your comments (Lines 179-186).

Results:
Line 159: In Lines 115-117, it suggests that women self-reported adhering to the OGTT process. Suggest saying, 'The majority of women reported consuming'. Self-reporting adherence to treatment regimens is not a reliable measure.

Line 161: As above, suggest rewording 'reported not drinking'.

Comments: We agree that self-reporting is not 100% reliable, but the CGM data showing increasing glucose provides some opportunity to confirm this. We have now added further information to address your comments.

Qualitative analysis:

Suggest including sub-themes that appear in tables under each heading (reassurance and convenience), with a brief description of each. Longer in-text quotes can be included in tables and more time can be spent describing each sub-theme.

Comments: We have now changed the text to include the subtheme headings with a brief description where necessary.

Discussion:
Line 299: What do these findings mean for other contexts, like low- and middle-income countries which also have large burdens of diabetes?

Comments: The use of CGM in GDM is still in early stages. We consider out-of-hospital testing to be a good option for women in the UK and internationally, especially where access to formal healthcare settings is difficult. We have now added further information to address your comments (Line 374-375)
